# On the expedient solution of the magneto-hydrodynamic Jeffery-Hamel flow of Casson fluid

**DOI:** 10.1038/s41598-018-34778-w

**Published:** 2018-11-05

**Authors:** S. S. Nourazar, A. Nazari-Golshan, F. Soleymanpour

**Affiliations:** 1 Mechanical Engineering Department, Amirkabir University of Technology, Tehran, Iran; 2 Physics Department, Shahed University, Tehran, Iran

## Abstract

The equation of magneto-hydrodynamic Jeffery-Hamel flow of non-Newtonian Casson fluid in a stretching/shrinking convergent/divergent channel is derived and solved using a new modified Adomian decomposition method (ADM). So far in all problems where semi-analytical methods are used the boundary conditions are not satisfied completely. In the present research, a hybrid of the Fourier transform and the Adomian decomposition method (FTADM), is presented in order to incorporate all boundary conditions into our solution of magneto-hydrodynamic Jeffery-Hamel flow of non-Newtonian Casson fluid in a stretching/shrinking convergent/divergent channel flow. The effects of various emerging parameters such as channel angle, stretching/shrinking parameter, Casson fluid parameter, Reynolds number and Hartmann number on velocity profile are considered. The results using the FTADM are compared with the results of ADM and numerical Range-Kutta fourth-order method. The comparison reveals that, for the same number of components of the recursive sequences over a wide range of spatial domain, the relative errors associated with the new method, FTADM, are much less than the ADM. The results of the new method show that the method is an accurate and expedient approximate analytic method in solving the third-order nonlinear equation of Jeffery-Hamel flow of non-Newtonian Casson fluid.

## Introduction

Jeffery-Hamel flow is commonly used as an important model problem for investigating various aspects of engineering applications such as mechanical, aerospace, chemical, civil, environmental and biomechanical science. Flow through rivers, channels and blood flow where arteries and capillaries are linked to each other are some examples of these applications. The applications of the Jeffery-Hamel equations, particularly in fluid mechanics, chemical and aerospace engineering received more elaborations recently. A few precise applications are chemical vapor deposition reactors, high-current arc in plasma generators, expanding or contracting regions in industrial machines, gas compressors and pipe sections. The problem of investigating the thermal radiation on classical Jeffery–Hamel flow problem, where the convergent/divergent channels are subjected to stretching or shrinking, which has important influence in the application to aerospace science and industry.

Jeffery^1^ and Hamel^2^ flows, describe the two dimensional, viscous, incompressible confined fluid flows between two planes at a specific diverging or converging angle. Rosenhead^3^ presented a general solution in terms of elliptic functions for this problem. Exact solutions for the thermal distributions of this flow when Millsaps and Pohlhausen^4^ found non-parallel plane walls held at a constant temperature. The importance of the study of Jeffery-Hamel problems comes from the fact that the fluid flow may be controlled by varying the intensity of the external magnetic field. The behavior of conducting fluids flowing in the presence of an external magnetic field is quite different than those when there is no magnetic field affecting the fluid flow^5,6^. The classical view of Jeffery-Hamel problems with an external magnetic field on a conducting fluid is studied by using the intensity of the magnetic field as a control parameter^5,6^. The study of stretching or shrinking sheets is an important area of interest because of its applications in many industries such as extrusion of polymer sheets from a die, the manufacturing of rubber sheets, glass fiber and paper production, etc. Crane^7^ did the first research on this subject. The steady two dimensional stagnation point flow towards a nonlinearly stretching surface was investigated by Zhu *et al*.^8^. Turkyilmazoglu^9^ considered the Jeffery–Hamel flow in stretchable convergent/divergent channels. His results indicate that stretching of the convergent and divergent channel amplifies the velocity profiles and an opposite effect in the case of shrinking resulting in back flow regions.

Casson fluid is one of the most popular non-Newtonian fluid models^10–13^. Some examples of the Casson fluid include chocolate, concentration juices, honey, jelly, and tomato sauce. Also blood through narrow arteries at low shear rate treated as Casson fluid^13^. Based on these applications, many authors studied various research problems with the case of Casson fluid. For example, Hayat *et al*.^14^ analyzed Soret and Dufour effects on magneto-hydrodynamic (MHD) flow of Casson fluid using homotopy analysis method (HAM).

The analytical and numerical solutions of Jeffery-Hamel problems are frequently reported using the numerical fourth-order Runge-Kutta method and the Adomian decomposition method (ADM)^9,15–27^. In all previous researches, the semi-analytical methods are used to solve the MHD Jeffery-Hamel problems without satisfying all boundary conditions in the entire domain of calculations. In the present study, it is intended to use a modified ADM that allows one to incorporate all boundary conditions into the solution. Then we would like to investigate and find out the effect of satisfying all boundary conditions on the results of the solution.

The main goal of the present study is to present an accurate and expedient approximate analytic solution of the third-order nonlinear equation of MHD Jeffery-Hamel flow of non-Newtonian Casson fluid. The Fourier transform is applied to the Adomian decomposition method, namely the FTADM^28^ in order to satisfy all boundary conditions in a wide range of spatial domain while this is not possible when applying the ordinary ADM. The FTADM, where all the boundary conditions are satisfied over the entire spatial domain is used in our calculations. The results using the FTADM are compared with those obtained by the ordinary ADM in order to investigate the effect of the incorporation of all boundary conditions on the results. The results using the FTADM are also compared with the results of numerical fourth-order Runge-Kutta method. Our comparison shows that, for the same number of components of the recursive equations, the results using the FTADM are much more accurate than the results of ordinary ADM. Moreover, to our knowledge no attempt is made so far to investigate the combined effect of Hartmann number and stretching/shrinking channel on non-Newtonian Casson fluid flow in the Jeffrey-Hamel (wedge) geometry. To this purpose, the mathematical formulation of the strongly nonlinear third-order MHD Jeffery-Hamel type equation of the non-Newtonian Casson fluid is derived and is solved by the FTADM. In non-Newtonian Casson fluid, we consider the interaction of conducting fluids and electromagnetic fluids. When a conducting fluid moves through a magnetic field consequently current may be induced and, in turn the current interacts with the magnetic field to produce a body force. When the conductor is either a liquid or gas, the electromagnetic force is then generated.

## Mathematical Formulation

**Figure 1:**
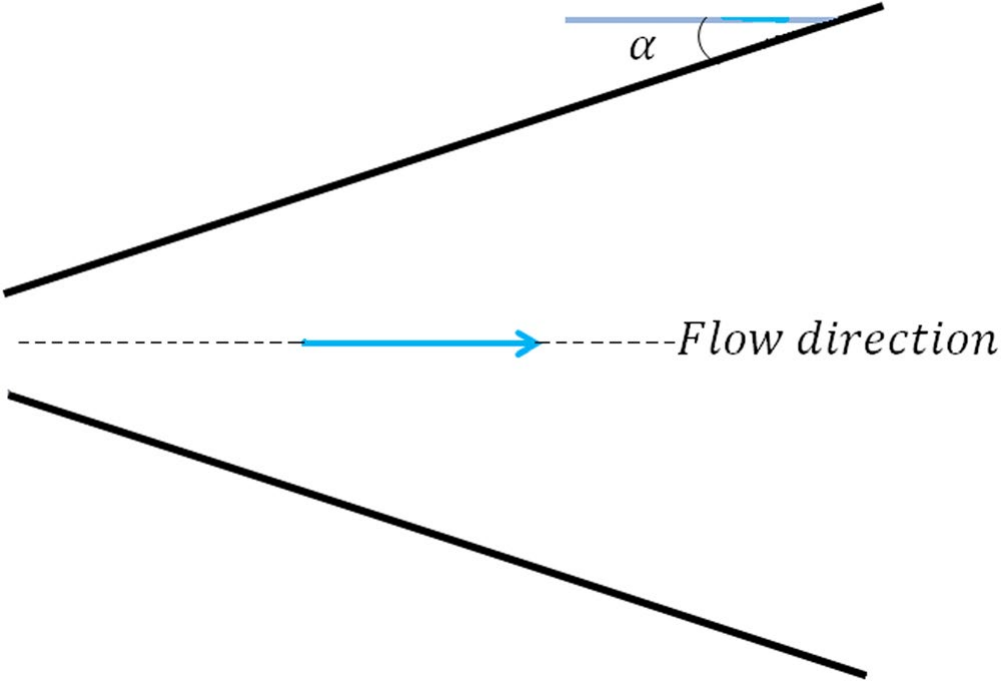
Geometry of the MHD Jeffery-Hamel flow.

We consider the two dimensional MHD flow of an incompressible non-Newtonian Casson fluid from a source or sink between two stretching or shrinking walls. Angle between the walls is 2*α*. Where *α* < 0, *α* > 0 represent the convergent walls and divergent walls, respectively. Figure (1) shows the geometry of the MHD Jeffery-Hamel flow. The walls stretch/shrink at a rate *s* with radial distance of *r* and the velocity at the walls is:

1 $$u={u}_{w}=\frac{s}{r}$$

The constitutive equation describing an incompressible non-Newtonian Casson fluid is written as follow^22^:

2 $${\tau }_{ij}=\{\begin{array}{cc}2({\mu }_{B}+\frac{{P}_{y}}{\sqrt{2\pi }}){e}_{ij} & \pi  > {\pi }_{c}\\ 2({\mu }_{B}+\frac{{P}_{y}}{\sqrt{2\pi }}){e}_{ij} & \pi  < {\pi }_{c}\end{array}\}\,\,$$

In Eq. (2), where *μ*_*B*_ denotes the plastic dynamic viscosity of the non-Newtonian fluid, *P*_*y*_ is the yield stress of the fluid, *π* is the product of the component of deformation rate with itself, namely, *π* = *e*_*ij*_*e*_*ij*_ where *e*_*ij*_ is the (*i*, *j*) th component of the deformation rate and *π*_*c*_ is a critical value of this product based on the non-Newtonian model.

We assume that the flow is purely radial and there is no magnetic field in the z-direction.

The conservation laws of mass and momentum for this problem are:

3 $$\nabla \cdot V=0$$

4 $$\rho (\frac{\partial V}{\partial t}+(V\cdot \nabla )V)=-\,\nabla p+\nabla \cdot {\tau }_{ij}+{f}_{B}$$

Consider the previously mentioned assumptions, Eqs (2–4) reduce to:

5 $$\frac{1}{r}\frac{\partial (ru)}{\partial r}=0$$

6 $$u\frac{\partial u}{\partial r}=-\,\frac{1}{\rho }\frac{\partial p}{\partial r}+\upsilon (1+\frac{1}{\,{\rm{\beta }}})(\frac{{\partial }^{2}u}{\partial {r}^{2}}+\frac{1}{r}\frac{\partial u}{\partial r}+\frac{1}{{r}^{2}}\frac{{\partial }^{2}u}{\partial {\theta }^{2}}-\frac{u}{{r}^{2}})-\frac{\sigma {B}_{0}^{2}}{\rho {r}^{2}}u$$

7 $$-\frac{1}{\rho r}\frac{\partial p}{\partial \theta }+\frac{2\upsilon }{{r}^{2}}(1+\frac{1}{{\rm{\beta }}})(\frac{\partial u}{\partial \theta })=0.$$

where, *u* is the velocity component in radial direction, *p* is the pressure, *υ* is kinematic viscosity, $\beta =\frac{{\mu }_{B}\sqrt{2\pi }}{{P}_{y}}$ is the Casson fluid parameter, *B*_0_ is the electromagnetic induction, *σ* is the conductivity of the fluid and *ρ* is the fluid density.

Associated boundary conditions for the present problem are:

8 $$\begin{array}{cc}{\rm{u}}={u}_{w}=\frac{s}{r} & {\rm{\theta }}=\pm \,{\rm{\alpha }}\,\end{array}$$

9 $$\begin{array}{cc}\frac{\partial {u}_{r}}{\partial \theta }=0,\,{\rm{u}}=\frac{{u}_{c}}{r}=U & {\rm{\theta }}=0.\end{array}$$

where, *u*_*c*_ is the centerline rate of movement, *u*_*w*_ is the velocity at the channel walls and *s* represent the stretching/shrinking rate.

From Eq. (5):

10 $$f(\theta )=ru$$

Using dimensionless parameters,

11 $$\begin{array}{cc}\,f(\eta )=\frac{f(\theta )}{{u}_{c}} & \eta =\frac{\theta }{\alpha }\end{array}$$

By eliminating the pressure terms from Eqs (6) and (7) and then using Eqs (10) and (11), we obtain the following third-order nonlinear differential equation:

12 $$(1+\frac{1}{\beta })\,f\prime\prime\prime (\eta )+2\alpha {\rm{Re}}f(\eta )f^{\prime} (\eta )+(4(1+\frac{1}{\beta })-H)\,{\alpha }^{2}f^{\prime} (\eta )=0,$$

Subject to the boundary conditions,

13 $$f(0)=1,\,f^{\prime} (0)=0,\,f(1)=C.$$

where *α* is the angle between the two planes, *β* is the Casson fluid parameter and $H=\sqrt{\frac{\sigma {{B}_{0}}^{2}}{\rho \upsilon }}$ is the Hartmann number.

The Reynolds number is:

14 $$\begin{array}{cc}Re\,=\frac{\alpha {u}_{c}}{\upsilon } & \,(\begin{array}{c}\alpha  > 0,\,U > 0:\,divergent\,channel\\ \alpha  < 0,\,U < 0:\,convergent\,channel\end{array})\end{array}$$

Values for skin friction coefficient, defined as:

15 $${c}_{f}=\frac{{\tau }_{r\theta }|{}_{\xi =1}}{\rho {U}^{2}}=\frac{1}{\mathrm{Re}}(1+\frac{1}{\beta })f^{\prime} (1)$$

## The Adomian Decomposition Method

The Adomian decomposition method (ADM) was created by Adomian^29–32^. The basic idea of the ADM for solving ordinary and partial differential equations is as follows. Assume a differential equation in the following form:

16 $$G(u)=g(t),$$

where *G* is an arbitrary operator. The operator *G* may generally be partitioned into two separate operators, the linear and the nonlinear operators as,

17 $$G(u)=L(u)+N(u)=g(t)$$

The unknown function *u*(*x*, *t*) of the linear operator can be decomposed by a series of solutions^33^:

18 $$u=\sum _{n={\rm{0}}}^{\infty }{u}_{n}.$$

Where the solution components *u*_*n*_, *n* ≥ 0, is calculated by the recursive equations. The linear operator, in light of Eq. (18), should be written as:

19 $$L(u)=L(\sum _{n={\rm{0}}}^{\infty }{u}_{n}).$$

However, the nonlinear term, in Eq. (17), may be expressed as an infinite series using the Adomian polynomials as^34–40^:

20 $$N(u)=\sum _{n={\rm{0}}}^{\infty }{A}_{n}=\sum _{n={\rm{0}}}^{\infty }\frac{{\rm{1}}}{n!}\frac{{d}^{n}}{d{\lambda }^{n}}{[N(\sum _{i={\rm{0}}}^{\infty }{\lambda }^{i}{u}_{i})]}_{\lambda ={\rm{0}}},\,n=0,\,1,\,2,\,\mathrm{...}$$

With the aid of some algebraic manipulations, Eq. (20) can be rewritten as:

21 $$\begin{array}{rcl}N(u) & = & N({u}_{{\rm{0}}})+(\sum _{n={\rm{0}}}^{\infty }{u}_{n})N^{\prime} ({u}_{{\rm{0}}})+\frac{{\rm{1}}}{2!}{(\sum _{n={\rm{0}}}^{\infty }{u}_{n})}^{{\rm{2}}}N^{\prime\prime} ({u}_{{\rm{0}}})+\frac{{\rm{1}}}{3!}{(\sum _{n={\rm{0}}}^{\infty }{u}_{n})}^{{\rm{3}}}N\prime\prime\prime ({u}_{{\rm{0}}})+\mathrm{...}\\  & = & N({u}_{{\rm{0}}})+(u-{u}_{{\rm{0}}})N^{\prime} ({u}_{{\rm{0}}})+\frac{{\rm{1}}}{2!}{(u-{u}_{{\rm{0}}})}^{{\rm{2}}}N^{\prime\prime} ({u}_{{\rm{0}}})+\frac{{\rm{1}}}{3!}{(u-{u}_{{\rm{0}}})}^{{\rm{3}}}N\prime\prime\prime ({u}_{{\rm{0}}})+\mathrm{...}\end{array}$$

It is worth mentioning that Eq. (21) is obtained using the Taylor series expansion of the function *u* in vicinity of a function *u*_0_ and not in vicinity of a point as is commonly used. Substituting Eq. (20) and Eq. (19) into Eq. (17) one may obtain the following,

22 $$L(\sum _{n={\rm{0}}}^{\infty }{u}_{n})+\sum _{n={\rm{0}}}^{\infty }{A}_{n}=g(t).$$

The components of *u* can be easily obtained by solving the recursive equations obtained from Eq. (22).

## Basic idea of the FTADM

Taking the Fourier transform of both sides of Eq. (22), we obtain^28^:

23 $$L(\sum _{n={\rm{0}}}^{\infty }{\mathop{u}\limits^{\frown {}}}_{n})+\sum _{n={\rm{0}}}^{\infty }{\mathop{A}\limits^{\frown {}}}_{n}=\mathop{g}\limits^{\frown {}}(\omega ).$$

where the Adomian polynomials, *A*_*n*_ are:

24 $$\begin{array}{rcl}{A}_{0} & = & N({u}_{{\rm{0}}}),\\ {A}_{1} & = & {u}_{{\rm{1}}}N^{\prime} ({u}_{{\rm{0}}}),\\ {A}_{2} & = & {u}_{{\rm{2}}}N^{\prime} ({u}_{{\rm{0}}})+\frac{{\rm{1}}}{2!}{{u}_{{\rm{1}}}}^{2}N^{\prime\prime} ({u}_{{\rm{0}}}),\\ {A}_{3} & = & {u}_{{\rm{3}}}N^{\prime} ({u}_{{\rm{0}}})+{u}_{{\rm{1}}}{u}_{{\rm{2}}}N^{\prime\prime} ({u}_{{\rm{0}}})+\frac{{\rm{1}}}{{\rm{3}}!}{{u}_{{\rm{1}}}}^{3}N\prime\prime\prime ({u}_{{\rm{0}}}),\\ {A}_{4} & = & {u}_{{\rm{4}}}N^{\prime} ({u}_{{\rm{0}}})+(\frac{{\rm{1}}}{2!}{{u}_{{\rm{2}}}}^{2}+{u}_{{\rm{1}}}{u}_{{\rm{3}}})N^{\prime\prime} ({u}_{{\rm{0}}})+\frac{{\rm{1}}}{{\rm{2}}!}{{u}_{{\rm{1}}}}^{2}{u}_{{\rm{2}}}N\prime\prime\prime ({u}_{{\rm{0}}})+\frac{{\rm{1}}}{{\rm{4}}!}{{u}_{{\rm{1}}}}^{4}{N}^{(iv)}({u}_{{\rm{0}}}).\end{array}$$

Using Eq. (23), we introduce the recursive relations as:

25 $$\begin{array}{rcl}L({\mathop{u}\limits^{\frown {}}}_{{\rm{0}}}) & = & \mathop{g}\limits^{\frown {}},\\ \sum _{i={\rm{1}}}^{\infty }L({\mathop{u}\limits^{\frown {}}}_{i})+\sum _{i={\rm{0}}}^{\infty }{\mathop{A}\limits^{\frown {}}}_{i} & = & {\rm{0}}{\rm{.}}\end{array}$$

The recursive relations, Eq. (25), may easily be solved:

26 $$\begin{array}{rcl}L({\mathop{u}\limits^{\frown {}}}_{{\rm{0}}}) & = & \mathop{g}\limits^{\frown {}},\\ L({\mathop{u}\limits^{\frown {}}}_{{\rm{1}}})+{\mathop{A}\limits^{\frown {}}}_{{\rm{0}}} & = & 0,\\ L({\mathop{u}\limits^{\frown {}}}_{{\rm{2}}})+{\mathop{A}\limits^{\frown {}}}_{{\rm{1}}} & = & 0,\\ L({\mathop{u}\limits^{\frown {}}}_{{\rm{3}}})+{\mathop{A}\limits^{\frown {}}}_{{\rm{2}}} & = & 0,\\ \vdots  &  & \\ L({\mathop{u}\limits^{\frown {}}}_{k})+{\mathop{A}\limits^{\frown {}}}_{k-{\rm{1}}}={\rm{0}}{\rm{.}} & = & 0,\end{array}$$

Using the Maple software package the recursive equations, Eq. (26), may easily be solved alternatively.

## Case Study of the Jeffery-Hamel problem

We solve the one-dimensional third-order nonlinear differential equation of the Jeffery-Hamel flow of non-Newtonian Casson fluid to demonstrate the effectiveness and the validity of the FTADM method, over the entire domain. The aforesaid equation in the one-dimensional case is written as:

27 $$(1+\frac{1}{\beta })f\prime\prime\prime (\eta )+2\alpha \,{\rm{Re}}\,f(\eta )f^{\prime} (\eta )+(4(1+\frac{1}{\beta })-H)\,{\alpha }^{2}f^{\prime} (\eta )=0.$$

Equation (27) is solved subject to the mixed set of Dirichlet and Neumann boundary conditions as:

28 $$f(0)=1,\,f^{\prime} (0)=0,\,f(1)=C.$$

Where, *C* is the stretching or shrinking parameter.

### The FTADM as an accurate approximate analytic method

Taking the Fourier transform of Eq. (27), we obtain the following:

29 $$(1+\frac{1}{\beta })F(f\prime\prime\prime (\eta ))+2\alpha \,{\rm{Re}}\,F(f(\eta )f^{\prime} (\eta ))+(4(1+\frac{1}{\beta })-H)\,{\alpha }^{2}F(f^{\prime} (\eta ))=0,$$

Where *F* stands for the Fourier transform. Using the method of integration by parts, the Fourier transform of each individual part in Eq. (29) takes the following form:

30 $$\begin{array}{rcl}F(f\prime\prime\prime (\eta )) & = & -f^{\prime\prime} (0)-i\omega f^{\prime} (0)+{\omega }^{2}f(0)-i{\omega }^{3}F(f(\omega )),\\ F(f(\eta )f^{\prime} (\eta )) & = & -\frac{1}{2}{{f}^{2}(\eta )|}_{\eta =0}+\frac{1}{2}i\omega F({f}^{2}),\\ F(f^{\prime} (\eta )) & = & -f(0)+i\omega F(f(\omega )).\end{array}$$

Upon substitution of Eq. (30) into Eq. (29), we have:

31 $$\begin{array}{c}(1+\frac{1}{\beta })(\,-\,f^{\prime\prime} (0)-i\omega f^{\prime} (0)+{\omega }^{2}f(0)-i{\omega }^{3}F(f(\omega )))\\ \,+\,2\alpha \,{\rm{Re}}(\,-\frac{1}{2}{{f}^{2}(\eta )|}_{\eta =0}+\frac{1}{2}i\omega F({f}^{2}))\\ \,+\,(4(1+\frac{1}{\beta })-H){\alpha }^{2}(\,-\,f(0)+i\omega F(f(\omega )))=0.\end{array}$$

Although the Neumann and Dirichlet boundary conditions are incorporated into Eq. (31), however we still need to obtain the value of *f* ″(0) in order to solve Eq. (31). We take the triple integral from the left side of Eq. (27) and taking into account the boundary condition *f*(0) = 1, one obtains^18,19^:

32 $$\begin{array}{c}(1+\frac{1}{\beta }){\int }_{0}^{1}{\int }_{0}^{\eta }{\int }_{0}^{\eta }f\prime\prime\prime (\eta )d\eta d\eta d\eta +2\alpha \,{\rm{Re}}{\int }_{0}^{1}{\int }_{0}^{\eta }{\int }_{0}^{\eta }f(\eta )f^{\prime} (\eta )d\eta d\eta d\eta \\ +(4(1+\frac{1}{\beta })-H){\alpha }^{2}{\int }_{0}^{1}{\int }_{0}^{\eta }{\int }_{0}^{\eta }f^{\prime} (\eta )d\eta d\eta d\eta =0.\end{array}$$

Using integration by parts, Eq. (32) may be rewritten as:

33 $$\begin{array}{c}(1+\frac{1}{\beta })(f(1)-f(0)-f^{\prime} (0)-\frac{1}{2}f^{\prime\prime} (0))+2\alpha \,{\rm{Re}}(\frac{1}{2}{\int }_{0}^{1}{\int }_{0}^{\eta }{f}^{2}(\eta )d\eta d\eta -{\frac{1}{4}{f}^{2}|}_{\eta =0})\\ +(4(1+\frac{1}{\beta })-H){\alpha }^{2}({\int }_{0}^{1}{\int }_{0}^{\eta }f(\eta )d\eta d\eta -\frac{1}{2}f(0))=0.\end{array}$$

Upon using the boundary conditions given in Eq. (28) and rearranging terms, we obtain the relation for *f* ″(0) as:

34 $$\begin{array}{rcl}f^{\prime\prime} (0) & = & 2(c-1)+(\frac{2\alpha \beta \mathrm{Re}}{\beta +1})({\int }_{0}^{1}{\int }_{0}^{\eta }{f}^{2}(\eta )d\eta d\eta -{\frac{1}{2}{f}^{2}|}_{\eta =0})\\  &  & +(4-\frac{\beta H}{\beta +1}){\alpha }^{2}(2{\int }_{0}^{1}{\int }_{0}^{\eta }f(\eta )d\eta d\eta -1).\end{array}$$

By substituting Eq. (34) into Eq. (31) and making use of boundary conditions defined by Eq. (28), we deduce the following:

35 $$\begin{array}{c}(1+\frac{1}{\beta })(\begin{array}{c}2(1-c)-(\frac{2\alpha \beta {\rm{Re}}}{\beta +1})({\int }_{0}^{1}{\int }_{0}^{\eta }{f}^{2}(\eta )d\eta d\eta -{\frac{1}{2}{f}^{2}|}_{\eta =0})\\ -(4-\frac{\beta H}{\beta +1}){\alpha }^{2}(2{\int }_{0}^{1}{\int }_{0}^{\eta }f(\eta )d\eta d\eta -1)\\ +{\omega }^{2}-i{\omega }^{3}F(f(\eta ))\end{array})\\ \,+2\alpha \,{\rm{Re}}(-\frac{1}{2}{f}^{2}(\eta )|{}_{\eta =0}+\frac{1}{2}i\omega F({f}^{2}(\eta )))\\ \,+(4(1+\frac{1}{\beta })-H){\alpha }^{2}(-\,1+i\omega F(f(\eta )))=0.\end{array}$$

By making use of Eqs (33) and (27), Eq. (35) may be rewritten as follow:

36 $$\begin{array}{c}(1+\frac{1}{\beta })(\begin{array}{c}2(1-c)-(\frac{2\alpha \beta \mathrm{Re}}{\beta +1})({\int }_{0}^{1}{\int }_{0}^{\eta }\sum _{n={\rm{0}}}^{\infty }{A}_{n}(\eta )d\eta d\eta -{\frac{1}{2}\sum _{n={\rm{0}}}^{\infty }{A}_{n}|}_{\eta =0})\\ -(4-\frac{\beta H}{\beta +1}){\alpha }^{2}(2{\int }_{0}^{1}{\int }_{0}^{\eta }\sum _{n={\rm{0}}}^{\infty }{f}_{n}(\eta )d\eta d\eta -1)\\ +{\omega }^{2}-i{\omega }^{3}\sum _{n={\rm{0}}}^{\infty }{\mathop{f}\limits^{\frown {}}}_{n}(\omega )\end{array})\\ \,+\,2\alpha \mathrm{Re}(-\frac{1}{2}{\sum _{n={\rm{0}}}^{\infty }{A}_{n}(\eta )|}_{\eta =0}+\frac{1}{2}i\omega \sum _{n={\rm{0}}}^{\infty }{\mathop{A}\limits^{\frown {}}}_{n}(\omega ))\\ \,+\,(4(1+\frac{1}{\beta })-H){\alpha }^{2}(\,-\,1+i\omega \sum _{n={\rm{0}}}^{\infty }{\mathop{f}\limits^{\frown {}}}_{n}(\omega ))=0.\end{array}$$

Where the hat symbol denotes the Fourier transform. Using Eq. (36), we construct the following recursive equations:

37 $$\begin{array}{l}-i{\omega }^{3}\,{\mathop{f}\limits^{\frown {}}}_{0}(\omega )+{\omega }^{2}=0,\\ -i{\omega }^{3}{\mathop{f}\limits^{\frown {}}}_{1}(\omega )+2(1-c)=0,\\ -(1+\frac{1}{\beta })(\begin{array}{c}(\frac{2\alpha \beta {\rm{Re}}}{\beta +1})({\int }_{0}^{1}{\int }_{0}^{\eta }{A}_{0}(\eta )d\eta d\eta -{\frac{1}{2}{A}_{0}|}_{\eta =0})\\ +(4-\frac{\beta H}{\beta +1}){\alpha }^{2}(2{\int }_{0}^{1}{\int }_{0}^{\eta }{f}_{0}(\eta )d\eta d\eta -1)+i{\omega }^{3}\,{\mathop{f}\limits^{\frown {}}}_{2}(\omega )\end{array})\\ +\,2\alpha \,{\rm{Re}}\,(-\frac{1}{2}{{A}_{0}(\eta )|}_{\eta =0}+\frac{1}{2}i\omega {\mathop{A}\limits^{\frown {}}}_{0}(\omega ))\\ +\,(4(1+\frac{1}{\beta })-H){\alpha }^{2}(\,-1+i\omega {\mathop{f}\limits^{\frown {}}}_{0}(\omega ))=0,\\ -\,(1+\frac{1}{\beta })(\begin{array}{c}(\frac{2\alpha \beta \,{\rm{Re}}}{\beta +1})\,({\int }_{0}^{1}{\int }_{0}^{\eta }{A}_{1}(\eta )d\eta d\eta -{\frac{1}{2}{A}_{1}|}_{\eta =0})\\ +(4-\frac{\beta H}{\beta +1}){\alpha }^{2}(2{\int }_{0}^{1}{\int }_{0}^{\eta }{f}_{1}(\eta )d\eta d\eta -1)+i{\omega }^{3}{\mathop{f}\limits^{\frown {}}}_{3}(\omega )\end{array})\\ +\,2\alpha \,{\rm{Re}}\,(-\frac{1}{2}{A}_{1}(\eta )|{}_{\eta =0}+\frac{1}{2}i\omega {\mathop{A}\limits^{\frown {}}}_{1}(\omega ))\\ +\,(4(1+\frac{1}{\beta })-H){\alpha }^{2}(-1+i\omega {\mathop{f}\limits^{\frown {}}}_{1}(\omega ))=0,\\ \cdot \\ \cdot \\ \cdot \\ \,\,-\,(1+\frac{1}{\beta })(\begin{array}{c}(\frac{2\alpha \beta \,{\rm{Re}}}{\beta +1})\,({\int }_{0}^{1}{\int }_{0}^{\eta }{A}_{k}(\eta )d\eta d\eta -{\frac{1}{2}{A}_{k}|}_{\eta =0})\\ +(4-\frac{\beta H}{\beta +1}){\alpha }^{2}(2{\int }_{0}^{1}{\int }_{0}^{\eta }{f}_{k}(\eta )d\eta d\eta -1)+i{\omega }^{3}{\mathop{f}\limits^{\frown {}}}_{k+2}(\omega )\end{array})\\ \,\,+\,2\alpha \,{\rm{Re}}\,(-\frac{1}{2}{{A}_{k}(\eta )|}_{\eta =0}+\frac{1}{2}i\omega {\mathop{A}\limits^{\frown {}}}_{k}(\omega ))\\ \,\,+\,(4(1+\frac{1}{\beta })-H){\alpha }^{2}(-\,1+i\omega {\mathop{f}\limits^{\frown {}}}_{k}(\omega ))=0,\end{array}$$

where ${\mathop{A}\limits^{\frown {}}}_{0}$, ${\mathop{A}\limits^{\frown {}}}_{1}$, ${\mathop{A}\limits^{\frown {}}}_{2}$, ${\mathop{A}\limits^{\frown {}}}_{3}$, …, ${\mathop{A}\limits^{\frown {}}}_{k}$ are the Fourier transforms of the respective Adomian polynomials. The recursive equation, Eq. (37), is solved alternatively by making use of the Maple software package. The procedures of the solution process are as follows: from the first recursive equation of Eq. (37) we obtain ${\mathop{f}\limits^{\frown {}}}_{0}$, then by taking the inverse Fourier transform we obtain *f*_0_. From the second recursive equation of Eq. (37) we obtain ${f}_{0}$, then by taking the inverse Fourier transform we obtain *f*_1_. Having the values of *f*_0_ and *f*_1_ and using the third recursive equation of Eq. (37) we obtain ${\mathop{f}\limits^{\frown {}}}_{2}$ then by taking the inverse Fourier transform we obtain *f*_2_ and so on. The solution may be written as follows:

38 $$\begin{array}{rcl}{f}_{0} & = & 1,\\ {f}_{1} & = & (c-1){x}^{2},\\ {f}_{2} & = & \frac{1}{12}(\frac{\alpha (c-1)(H\alpha \beta -2\,{\rm{Re}}\beta -4\alpha \beta -4\alpha )}{\beta +1}){x}^{4}\\  &  & -\frac{1}{12}(\frac{\alpha (c-1)(H\alpha \beta -2\,{\rm{Re}}\beta -4\alpha \beta -4\alpha )}{\beta +1}){x}^{2},\\ {f}_{3} & = & \frac{1}{720}\frac{1}{{(\beta +1)}^{2}}(\begin{array}{c}(2{H}^{2}{\alpha }^{3}{\beta }^{2}-8H\,{\rm{Re}}{\alpha }^{2}{\beta }^{2}-16H{\alpha }^{3}{\beta }^{2}\\ -16H{\alpha }^{3}\beta +8\,{{\rm{Re}}}^{2}\alpha {\beta }^{2}+32\,{\rm{Re}}{\alpha }^{2}{\beta }^{2}+32{\alpha }^{3}{\beta }^{2}\\ +32\,{\rm{Re}}{\alpha }^{2}\beta -24\,{\rm{Re}}{\beta }^{2}c+64{\alpha }^{3}\beta \\ +24\,{\rm{Re}}{\beta }^{2}-24\,{\rm{Re}}\beta c+32{\alpha }^{3}+24\,{\rm{Re}}\beta )\alpha (c-1)\end{array}){x}^{6}\\  &  & +\,\frac{1}{720}\frac{1}{{(\beta +1)}^{2}}(\begin{array}{c}(-\,5{H}^{2}{\alpha }^{3}{\beta }^{2}+20H\,{\rm{Re}}{\alpha }^{2}{\beta }^{2}\\ +40H{\alpha }^{3}{\beta }^{2}+40H{\alpha }^{3}\beta -20\,{{\rm{Re}}}^{2}\alpha {\beta }^{2}\\ -80\,{\rm{Re}}{\alpha }^{2}{\beta }^{2}-80{\alpha }^{3}{\beta }^{2}\\ -80\,{\rm{Re}}{\alpha }^{2}\beta -160{\alpha }^{3}\beta -80{\alpha }^{3})\alpha (c-1)\end{array}){x}^{4},\\  &  & +\,\frac{1}{720}\frac{1}{{(\beta +1)}^{2}}(\begin{array}{c}(3{H}^{2}{\alpha }^{3}{\beta }^{2}-12H\,{\rm{Re}}{\alpha }^{2}{\beta }^{2}\\ -24H{\alpha }^{3}{\beta }^{2}-24H{\alpha }^{3}\beta \\ +12\,{{\rm{Re}}}^{2}\alpha {\beta }^{2}+48\,{\rm{Re}}{\alpha }^{2}{\beta }^{2}\\ +48{\alpha }^{3}{\beta }^{2}+48\,{\rm{Re}}{\alpha }^{2}\beta +24\,{\rm{Re}}{\beta }^{2}c\\ +96{\alpha }^{3}\beta -24\,{\rm{Re}}{\beta }^{2}\\ +24\,{\rm{Re}}\beta c+48{\alpha }^{3}-24\,{\rm{Re}}\beta )\alpha (c-1)\end{array}){x}^{2},\end{array}$$

## Results and Discussion

The objective of the present study is to incorporate all boundary conditions into our solution by apply the Fourier transform and Adomian decomposition method to obtain an explicit analytical solution of the Jeffery-Hamel flow of Casson fluid. The results of the solution are compared with the Adomian decomposition method and the numerical method using the Runge-Kutta fourth-order method.

In order to show the validity and accuracy of the results of our method, we compare the results of Abbasbandy^5^ obtained via the exact solution and the numerical Runge-Kutta fourth-order method. Tables (1) and (2) show the comparison between the present results of *f* ″(0) with the exact solutions and numerical results of Abbasbandy^5^ for six different Hartmann numbers when Re = 10, *β* = ∞, *C* = 0 and *α* = ±5°, respectively, (*C* = 0 corresponds to the stationary rigid walls condition and *β* = ∞ relates to the simple Newtonian fluid model.) where the angle *α* is converted from the unit of degrees to the unit of radians. The Hartmann number is dependent only on the strength of the applied magnetic field and on the properties of the fluid, thereby the increase in the Hartmann number with increase in the strength of magnetic field may be increased the stability of MHD flows. From Tables (1) and (2) it is clear that there is an excellent agreement between the present results (FTADM), the exact solutions and numerical results of Abbasbandy^5^. Table 3 shows comparison of the present results of velocity obtained from the FTADM with the numerical Runge-Kutta fourth-order results for convergent and divergent channels.

**Table 1:** Comparison between the present results of *f* ″(0) with the exact and numerical results of Abbasbandy^5^ for different values of Hartmann numbers of *α* = +5°, *β* = ∞, *Re* = 10 and *C* = 0.

Hartmann number	Numerical Results^5^	Exact^5^	Present results
0	−2.25194858	−2.251948602981818	−2.25194851
200	−1.98460616	−1.984606164603458	−1.98460618
400	−1.75409306	−1.754093033347798	−1.75409301
600	−1.55460600	−1.554605992057426	−1.55460620
800	−1.38137005	−1.381369953213575	−1.38137040
1000	−1.23043721	−1.230437181792459	−1.23043729

**Table 2:** Comparison between the present results of *f* ″(0) with the exact and numerical results of Abbasbandy^5^ for different values of Hartmann numbers of *α* = −5°, *β* = ∞, *Re* = 10 and *C* = 0.

Hartmann number	Numerical Results^5^	Exact^5^	Present results
0	−1.78454677	−1.784546840578866	−1.78454677
200	−1.57612994	−1.576129888848735	−1.57612997
400	−1.39631757	−1.396317528806288	−1.39631749
600	−1.24053452	−1.240534453880220	−1.24053448
800	−1.10504860	−1.105048380482003	−1.10504980
1000	−0.98679397	−0.986793975627043	−0.98679393

**Table 3:** Comparison between the numerical and the FTADM results for velocity of *β* = 0.5, *Re* = 10, *H* = 100, *C* = 0.4 and *α* = ±5°.

_*η*_	*α* = +5°			α = −5°		
	_Numerical_	_FTADM_	Error	_Numerical_	_FTADM_	Error
0	1.0000000000	1.0000000000	0.00E + 00	1.000000000	1.000000000	0.00E + 00
0.1	0.9938558387	0.9938558390	3.02E − 10	0.9943517131	0.9943517129	2.01E − 10
0.2	0.9754451486	0.9754451488	2.05E − 10	0.9773615295	0.9773615301	6.14E − 10
0.3	0.9448319217	0.9448319218	1.06E − 10	0.9488942412	0.9488942416	4.22E − 10
0.4	0.9021182040	0.9021182039	1.11E − 10	0.9087270814	0.9087270811	3.3E − 10
0.5	0.8474372786	0.8474372782	4.72E − 10	0.8565537726	0.8565537728	2.33E − 10
0.6	0.7809442987	0.7809442992	6.4E − 10	0.7919905477	0.7919905480	3.79E − 10
0.7	0.7028045214	0.7028045213	1.42E − 10	0.7145844935	0.7145844930	7E − 10
0.8	0.6131793066	0.6131793063	4.89E − 10	0.6238246612	0.6238246616	6.41E − 10
0.9	0.5122100270	0.5122100269	1.95E − 10	0.5191564703	0.5191564695	1.54E − 09
1.0	0.4000000000	0.4000000000	0.00E + 00	0.4000000000	0.4000000000	0.00E + 00

**Figure 2:**
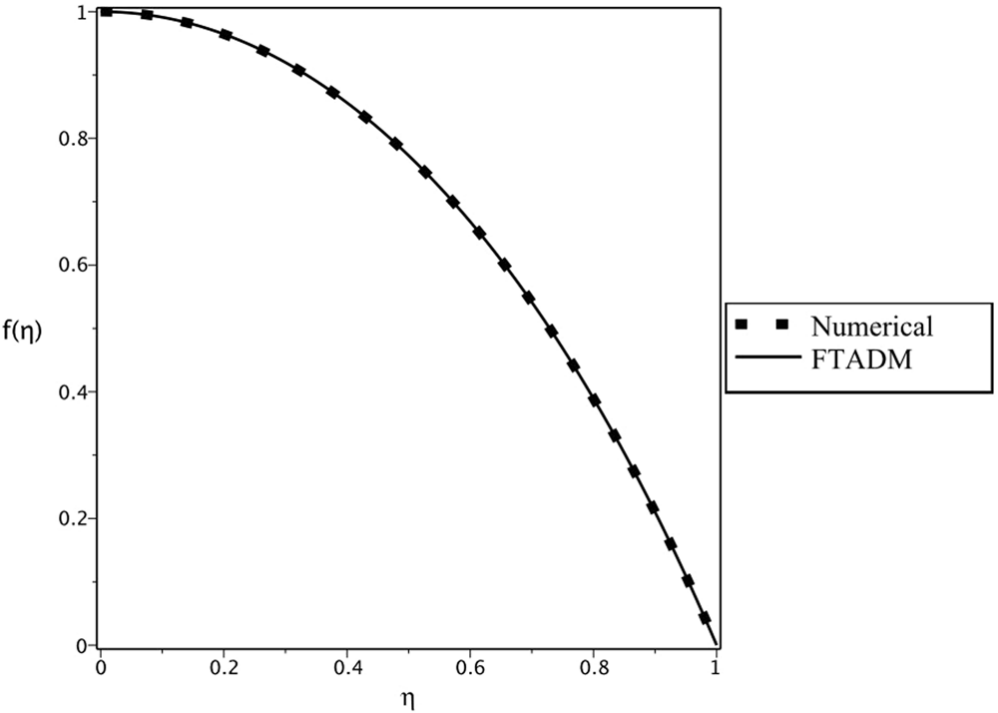
Comparison of numerical and FTADM results for the velocity profile of *Re* = 10, *α* = 5° *β* = 0.5, *H* = 800 and *C* = 0.

In addition, Fig. 2 displays comparison of the FTADM results and the numerical Runge-Kutta fourth-order results for Re = 10, *α* = 5°, *β* = 0.5, *H* = 800, *C* = 0 graphically. Our comparison shows excellent agreement with the numerical Runge-Kutta results. Table 4 gives a comparison of the convergence rate of the FTADM at different number of truncated terms (N) against the numerical approximations. The relative errors given in Table 4, is evaluated as follow:

$$Error=|\frac{f{(\eta )}_{Numerical}-f{(\eta )}_{FTADM}}{f{(\eta )}_{Numerical}}|$$

**Table 4:** Comparison between the numerical and the FTADM results for velocity at different recursive components, *N*, with *Re* = 10, *α* = 5°, *β* = 0.5, *H* = 100 and *C* = 0.

*η*	*N*			Numerical solution	Relative Error *N* = 4	Relative Error *N* = 10
	4	7	10			
0.1	0.9898008784	0.9897996972	0.9897996987	0.9897996987	1.19E − 06	0.00E + 00
0.2	0.9592394570	0.9592347299	0.9592347358	0.9592347355	4.92E − 06	3.13E − 10
0.3	0.9084197532	0.9084091756	0.9084091889	0.9084091885	1.16E − 05	4.40E − 10
0.4	0.8375024975	0.8374840878	0.8374841095	0.8374841089	2.20E − 05	7.16E − 10
0.5	0.7466864463	0.7466591195	0.7466591497	0.7466591496	3.66E − 05	1.34E − 10
0.6	0.6361827532	0.6361472572	0.6361472936	0.6361472936	5.57E − 05	0.00E + 00
0.7	0.5061829202	0.5061429849	0.5061430222	0.5061430211	7.88E − 05	2.17E − 09
0.8	0.3568210138	0.3567841843	0.3567842205	0.3567842141	1.03E − 04	1.79E − 08
0.9	0.1881309742	0.1881079232	0.1881079620	0.1881079402	1.22E − 04	1.16E − 07

A reasonable trend of approach and excellent agreement of our solution with the numerical solution are evident. Table 5 shows a comparison between relative errors of analytical solutions obtained by the ADM and the FTADM methods. It is evident from Table 5 that for the same number of truncated terms (N = 6), the maximum relative error associated with the ADM is in the order of 10^−4^, while the maximum relative error associated with the FTADM is in the order of 10^−8^.

**Table 5:** Comparison between the ADM and the FTADM results for velocity of *α* = 5°, *β* = ∞, *Re* = 50, *H* = 100, *C* = 0 and 6 number of different recursive components, *N*.

*η*	*N* = 6		Numerical results	ADM Relative error	FTADM Relative error
	ADM	FTADM			
0.1	0.9834997373	0.9835024682	0.9835024694	2.78E-06	1.22E-09
0.2	0.9352862085	0.9352969198	0.9352969242	1.15E-05	4.70E-09
0.3	0.8589889554	0.8590123298	0.8590123364	2.72E-05	7.68E-09
0.4	0.7599496556	0.7599896058	0.7599896153	5.26E-05	1.25E-08
0.5	0.6443653263	0.6444250034	0.6444250209	9.26E-05	2.72E-08
0.6	0.5184118824	0.5184938988	0.5184939138	1.58E-04	2.89E-08
0.7	0.3875188487	0.3876252333	0.3876252462	2.74E-04	3.33E-08
0.8	0.2558885925	0.2560173787	0.2560173664	5.03E-04	4.80E-08
0.9	0.1262774635	0.1264016134	0.1264016241	9.82E-04	8.47E-08

**Figure 3:**
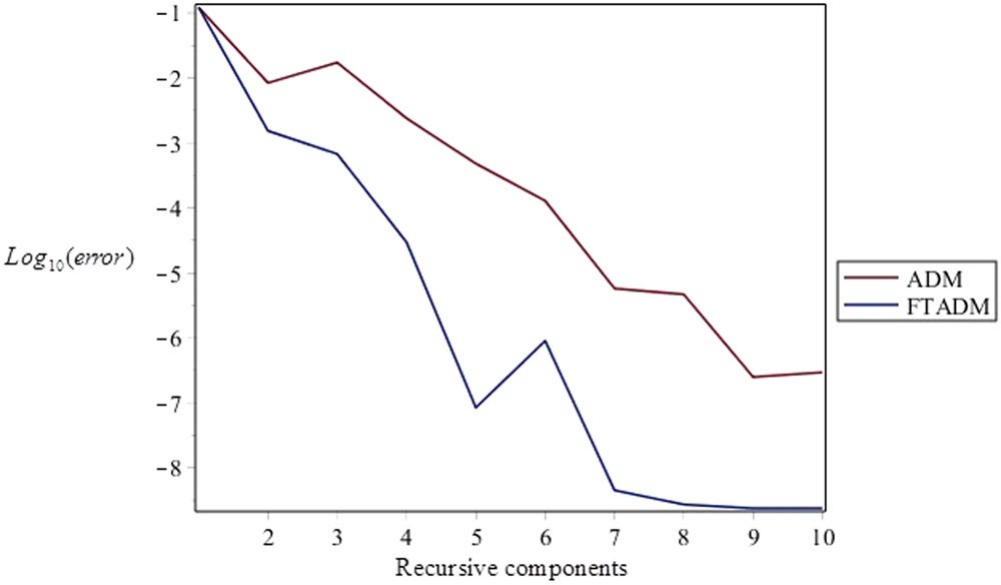
Comparison of errors for the results of velocity using ADM and FTADM at different recursive components of *Re* = 50, *α* = 5°, *β* = 0.5, *H* = 100 and *C* = 0.

Figure 3 depicts the comparison of the errors of ADM with FTADM results of velocity for different recursive components. According to this figure, the errors associated with the FTADM are much less than the ordinary ADM.

Table 6 displays the comparison between the numerical and the FTADM values of *f*′(1) and skin friction coefficient versus different values of Hartmann number, Reynolds number, angle *α*, stretching/shrinking parameter and Casson fluid parameter. The comparison shows an excellent agreement between our results using the FTADM and the ones obtained from the numerical Runge-Kutta fourth-order method.

**Table 6:** Comparison between the numerical and the FTADM values of skin friction coefficient for different values of parameters.

*α* ^0^	*β*	*Re*	*H*	*C*	*f* ′(1) Numerical	*f* ′(1) FTADM	*C*_*f*_ Re FTADM
+5	0.5	10	0	−2	−6.043227584	−6.043227630	−18.129682890
+5	0.5	10	0	−1.6	−5.197412525	−5.197412555	−15.592237665
+5	0.5	10	0	−1.2	−4.363747577	−4.363747596	−13.091242788
+5	0.5	10	0	−0.8	−3.542325292	−3.542325301	−10.626975903
+5	0.5	10	0	−0.4	−2.733239988	−2.733239989	−8.199719967
+5	0.5	10	0	0	−1.936587792	−1.936587793	−5.809763379
+5	0.5	10	0	0.4	−1.152466701	−1.152466702	−3.457400106
+5	0.5	10	0	0.8	−0.3809767939	−0.3809766346	−1.142929904
+5	0.5	10	0	1.2	0.3777802578	0.3777805083	1.133341525
+5	0.5	10	0	1.6	1.123700786	1.123700786	3.371102358
+5	0.5	10	0	2	1.856678155	1.856678154	5.570034462
−5	0.5	10	0	−1.5	−4.987645201	−4.987645225	−14.962935675
−5	0.5	10	0	−0.5	−3.050736481	−3.050736488	−9.152209464
−5	0.5	10	0	0	−2.053021018	−2.053021019	−6.159063057
−5	0.5	10	0	0.5	−1.036048173	−1.036048173	−3.108144519
−5	0.5	10	0	1.5	1.054945633	1.054945633	3.164836899
−5	0.5	10	0	2	2.128614899	2.128614902	6.385844706
+5	0.5	10	400	0	−2.104564342	−2.104564345	−6.313693035
+5	0.5	10	800	0	−2.267147185	−2.267147197	−6.801441591
+5	0.5	10	1000	0	−2.346529243	−2.346529269	−7.039587807
−5	0.5	10	400	0	−2.218230920	−2.218230920	−6.654692760
−5	0.5	10	1000	0	−2.456229991	−2.456229999	−7.368689997
−5	0.5	10	2000	0	−2.829543755	−2.829545358	−8.488636074
+5	0.5	25	0	0	−1.848698106	−1.848698117	−5.546094351
+5	0.5	50	0	0	−1.701360965	−1.701346693	−5.104040079
−5	0.5	25	0	0	−2.139695966	−2.139695971	−6.419087913
−5	0.5	50	0	0	−2.282747697	−2.282747117	−6.848241351
+5	1	10	0	0	−1.907340664	−1.907340671	−3.814681342
+5	1.5	10	0	0	−1.889768046	−1.889768052	−3.149613420
+5	2	10	0	0	−1.878043187	−1.878043192	−2.817064788
+5	∞	10	0	0	−1.819308257	−1.819311691	−1.819311691
−5	0.3	10	0	0	−2.035169802	−2.035169804	−8.819069151
−5	0.5	10	0	0	−2.053021018	−2.053021019	−6.159063057
−5	∞	10	0	0	−2.168452125	−2.168452170	−2.168452170
+1	0.3	100	0	0	−1.919026569	−1.919030265	−8.315797815
+3	0.3	100	0	0	−1.754690644	−1.754592253	−7.603233096
+5	0.3	100	0	0	−1.587507172	−1.587506417	−6.879194474
−1	0.3	100	0	0	−2.080121413	−2.080121377	−9.013859300
−3	0.3	100	0	0	−2.237674926	−2.237674787	−9.696590744
−5	0.3	100	0	0	−2.391472070	−2.391464344	−10.363012157

**Figure 4:**
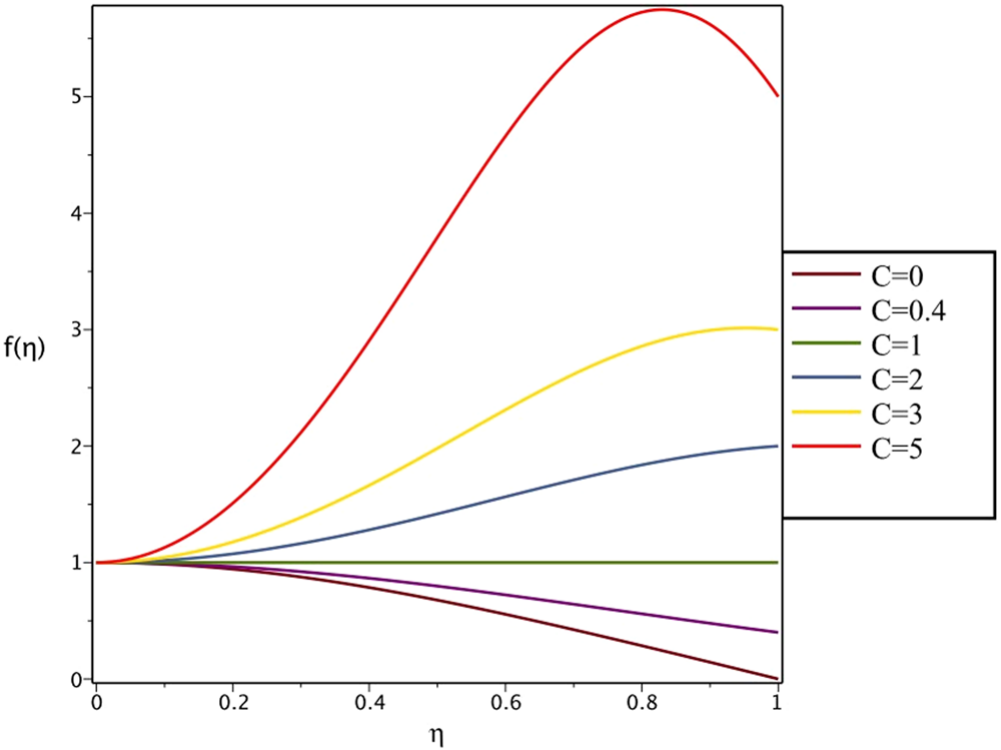
Effect of variations of *C* on the velocity profile in divergent channel of *Re* = 100, *α* = 5°, *β* = 0.5 and *H* = 100.

**Figure 5:**
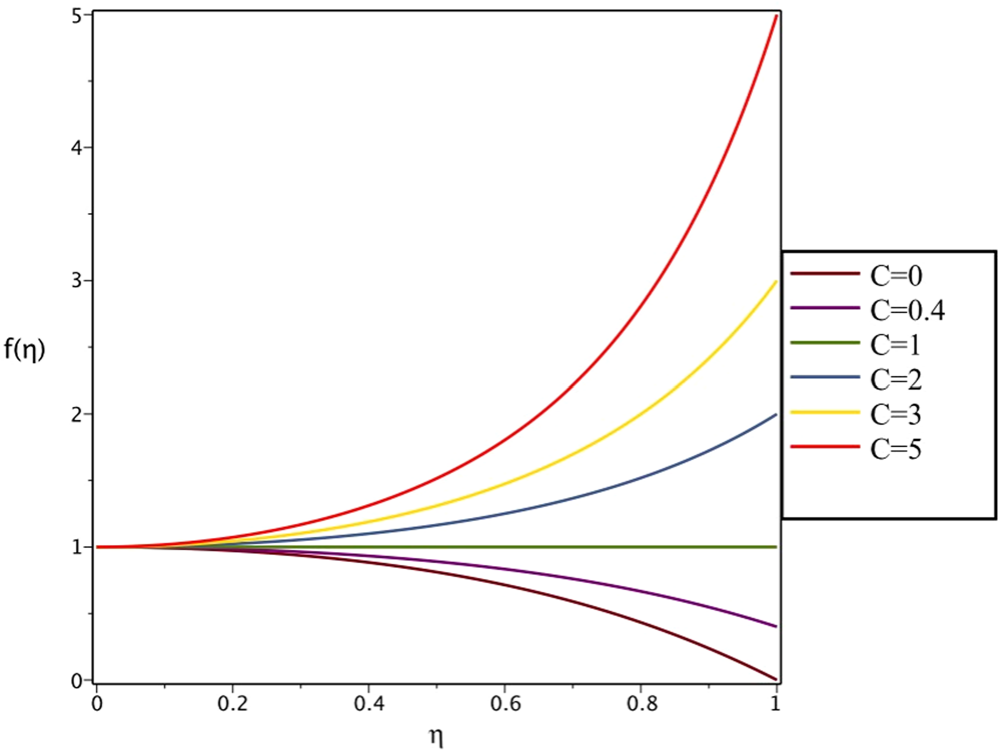
Effect of variations of *C* on the velocity profile in convergent channel of *Re* = 100, *α* = −5°, *β* = 0.5 and *H* = 100.

**Figure 6:**
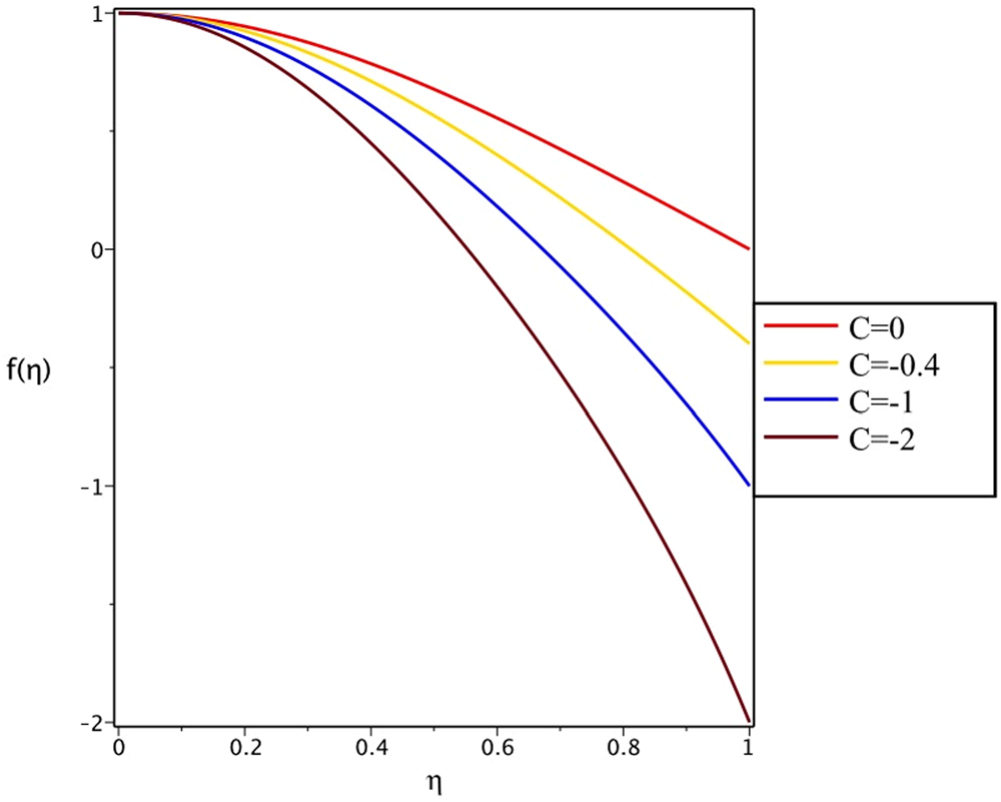
Effect of variations of *C* on the velocity profile in divergent channel of *Re* = 100, *α* = 5°, *β* = 0.5 and *H* = 100.

**Figure 7:**
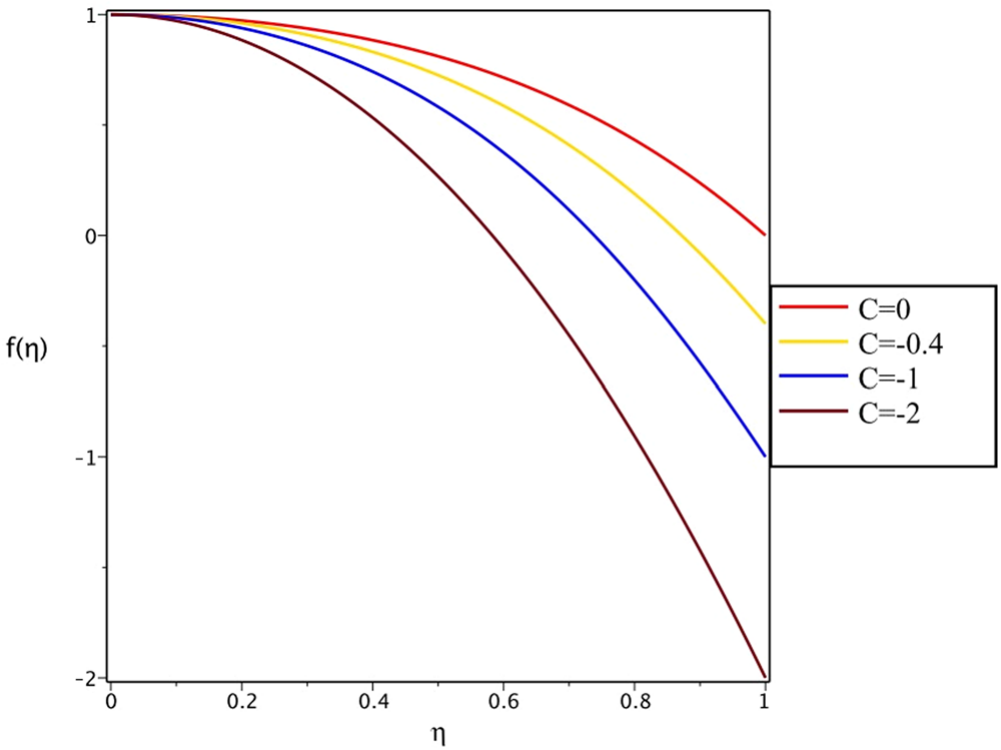
Effect of variations of *C* on the velocity profile in convergent channel of *Re* = 100, *α* = −5°, *β* = 0.5 and *H* = 100.

**Figure 8:**
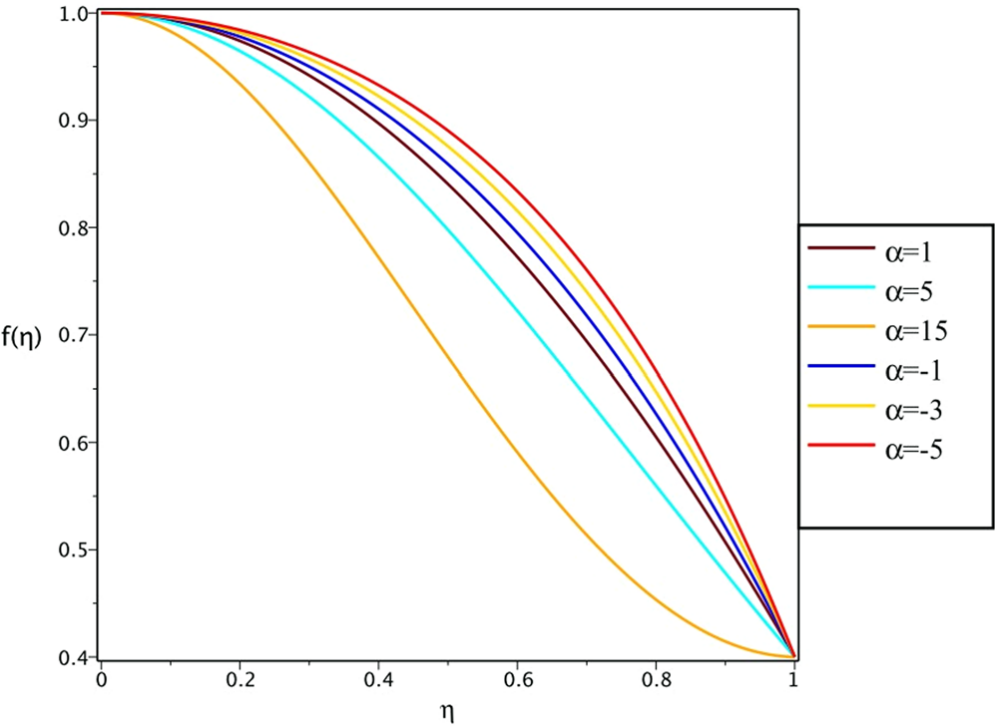
Effect of variations of *α* on the velocity profile in stretching divergent/convergent channel of *Re* = 100, *β* = 0.5, *H* = 100 and *C* = 0.4.

**Figure 9:**
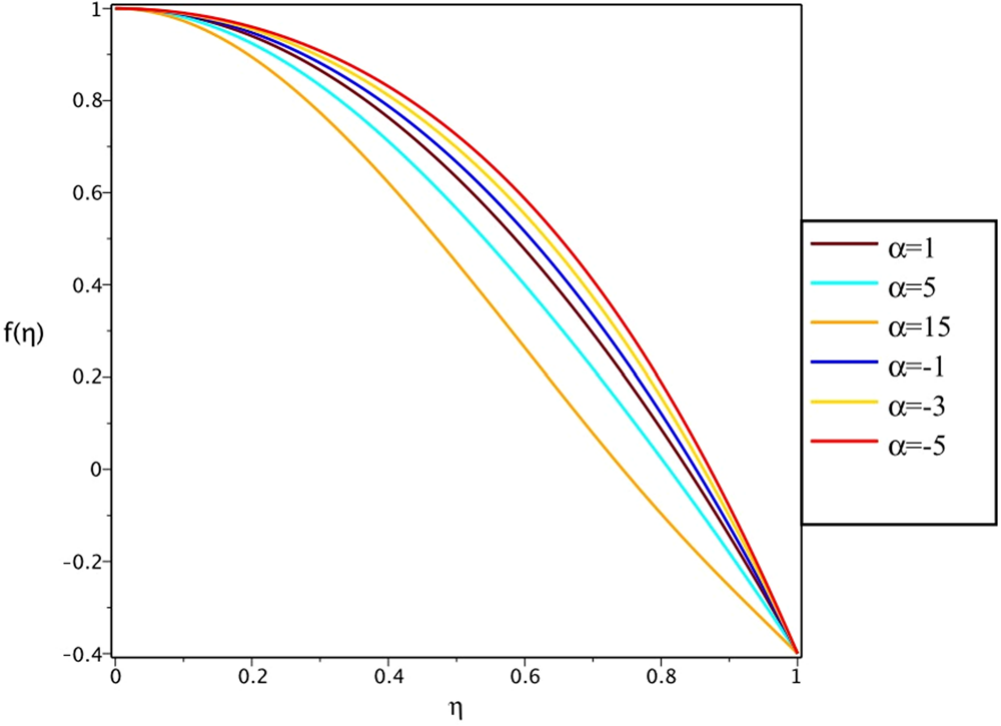
Effect of variations of *α* on the velocity profile in shrinking divergent/convergent channel of *Re* = 100, *β* = 0.5, *H* = 100 and *C* = −0.4.

**Figure 10:**
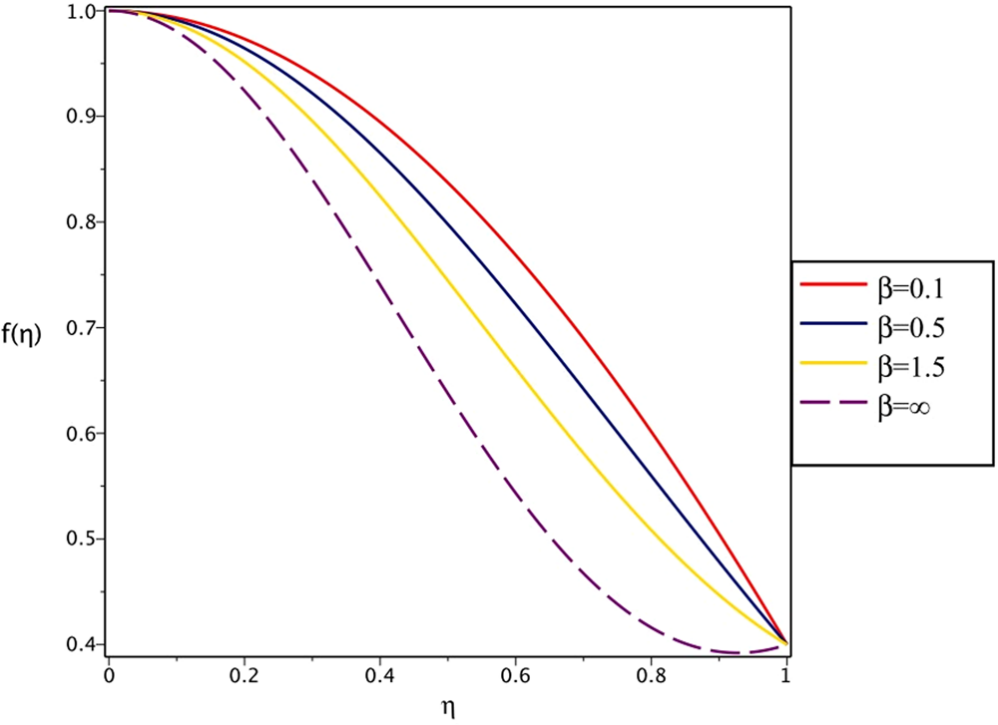
Effect of variations of *β* on the velocity profile in divergent channel of *Re* = 100, *α* = 5°, *H* = 100 and *C* = 0.4.

**Figure 11:**
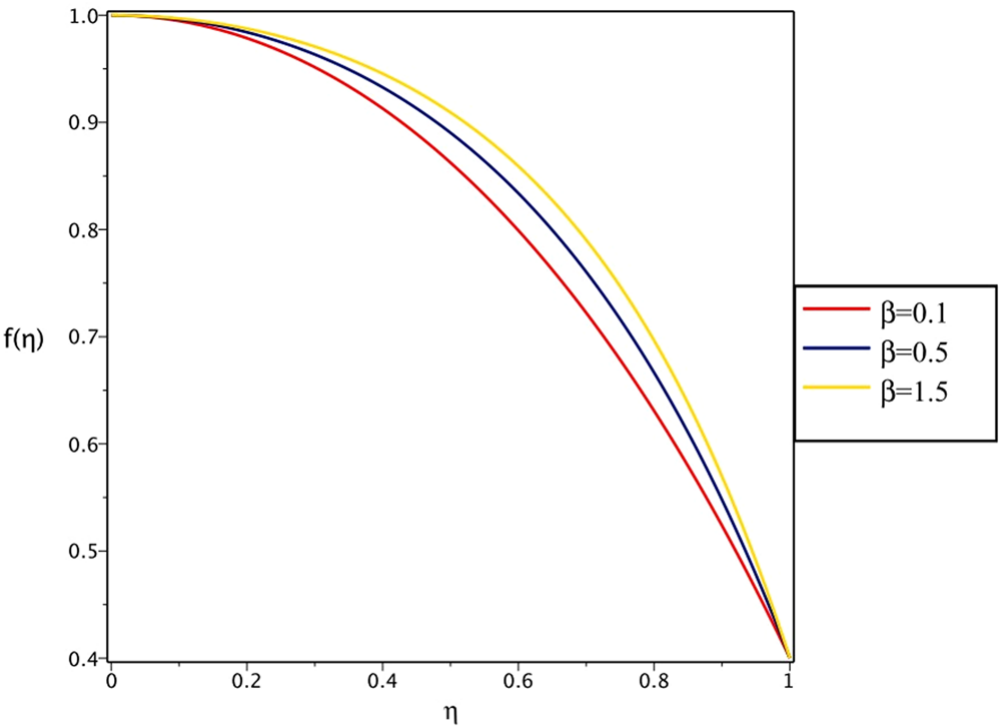
Effect of variations of *β* on the velocity profile in convergent channel of *Re* = 100, *α* = −5°, *H* = 100 and *C* = 0.4.

**Figure 12:**
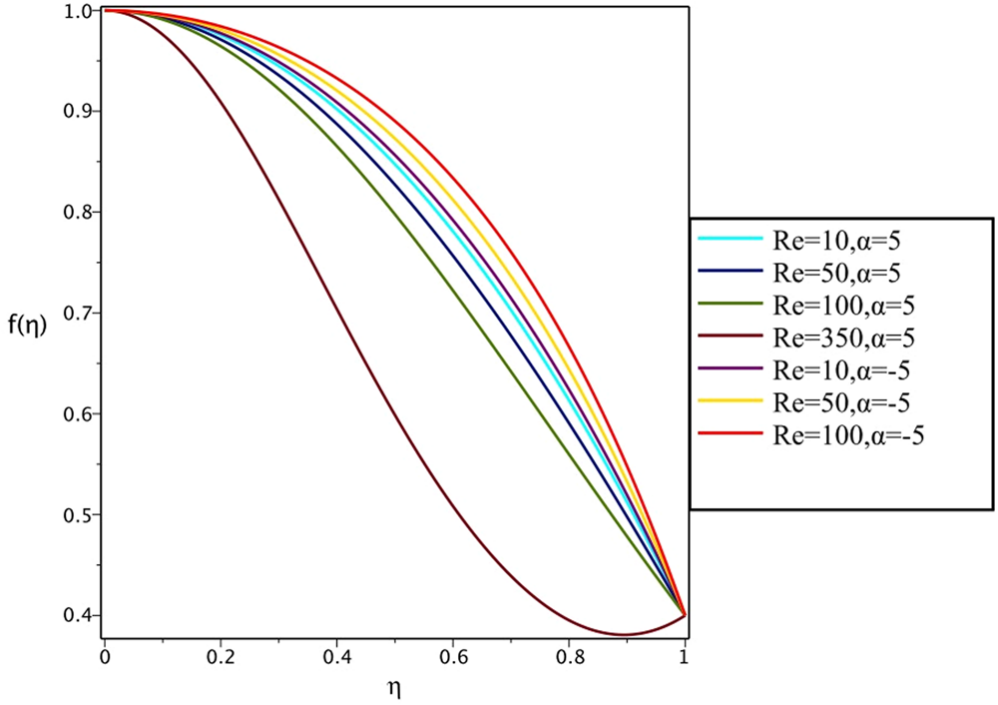
Effect of variations of *Re* on the velocity profile in stretching channel of *α* = ±5°, *β* = 0.5, *H* = 100 and *C* = 0.4.

**Figure 13:**
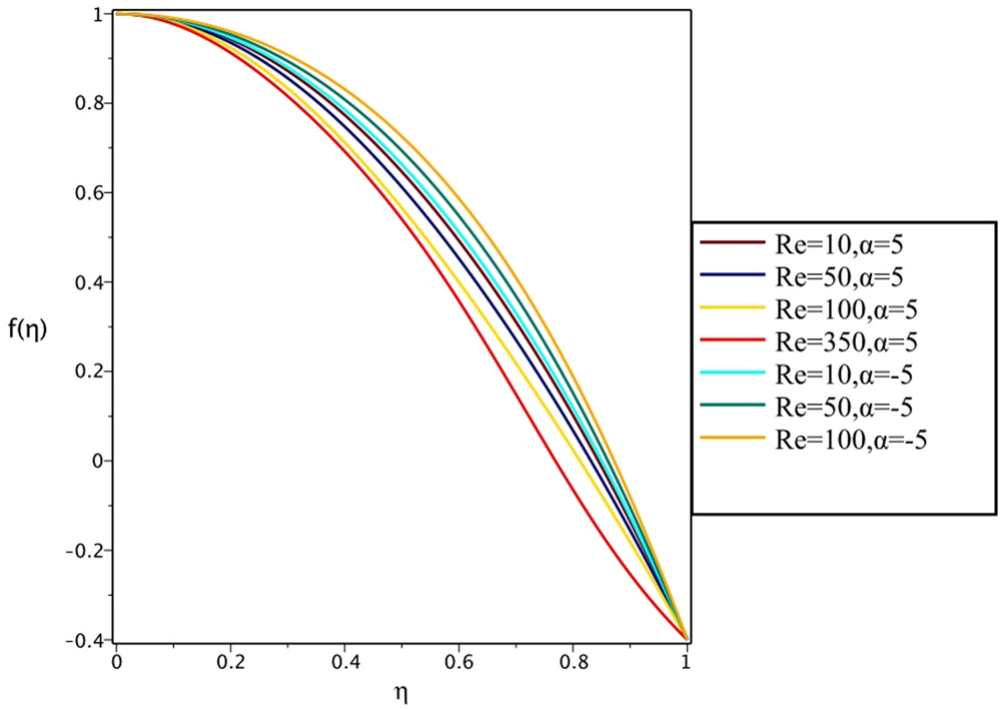
Effect of variations of *Re* on the velocity profile in shrinking divergent/convergent channel of *α* = ±5°, *β* = 0.5, *H* = 100 and *C* = −0.4.

Figures 4, 5, 6 and 7 demonstrate the effect of stretching/shrinking parameter on the fluid velocity profile in the convergent and divergent channel, respectively. It is clear that for stretching channel, velocity profile increases with the increasing of stretching parameter. In the shrinking channel, the velocity profile decreases due to an increase in the absolute value of shrinking parameter. In the case of stretching divergent channel, with increasing stretching parameter or increasing Reynolds number the velocity increases. This nonlinear increment in velocity is probably due to more drag force acting on the plate at large values of stretching parameter. Figures 8 and 9 depict the variations of the fluid velocity with an opening angle for stretching/shrinking divergent/convergent channels. According to these figures, the velocity profiles act as a decreasing function of opening angle for stretching/shrinking divergent channel. In the stretching/shrinking convergent channel, velocity profile increases with the increase of absolute value of opening angle. Figures 10 and 11 illustrate the effect of Casson fluid parameter on the fluid velocity profiles in the divergent and convergent channels, respectively. In the divergent channel, as the Casson fluid parameter increases the velocity profiles decrease significantly. On the other word, Fig. 10 shows that higher values of Casson fluid parameter have the tendency to decelerate the velocity of fluid flow. It is expected that an increase in Casson fluid parameter is used to decrease the stress that increases the value of dynamic viscosity, thereby produce a resistance in the fluid flow. It is interesting to mention that when the Casson fluid parameter increases indefinitely, the problem reduces to a Newtonian fluid case. In the convergent channel, an opposite behavior is observed. The effect of Reynolds number on the fluid velocity is shown in Figs 12 and 13. The graphs show that in stretching/ shrinking divergent channels, as the Reynolds number increases the fluid velocity decreases. An opposite trend is seen for stretching/shrinking convergent channels, where the velocity is an increasing function of Reynolds number.

## Conclusions

- In the present work, a new modification of the ADM, the FTADM, is proposed in order to incorporate the boundary conditions into our solution of MHD Jeffery-Hamel flow of non-Newtonian Casson fluid. Where the boundary conditions may not be incorporated into solution when the ADM is used.
- The comparison between our velocity results and *f* ″(0) at different Hartmann numbers obtained by using the FTADM with the exact and numerical results of Abbasbandy^5^ for the MHD Jeffery-Hamel flow of Newtonian fluid shows excellent agreement.
- The results obtained by the FTADM of divergent and convergent channels for the same number of components of the recursive sequence are compared with those obtained by the ADM. The comparison shows that the relative errors associated with the FTADM are much less than the ADM.
- We conclude that FTADM is more accurate than ADM and therefore the FTADM is an effective and expedient approximate semi-analytical method for solving the nonlinear equation of MHD Jeffery-Hamel flow of non-Newtonian Casson fluid.
- Our results show that in the case of stretching divergent channel, with increasing stretching parameter or increasing Reynolds number the velocity increases. This nonlinear increment in velocity is probably due to more drag force acting on the plate at large values of stretching parameter.

## Electronic supplementary material

Supplementary Information
