# Supplementary material for: On the expedient solution of the magneto-hydrodynamic Jeffery-Hamel flow of Casson fluid
Source: Sci Rep. 2018 Nov 5;8:16358. doi: 10.1038/s41598-018-34778-w (PMC6218494; doi:10.1038/s41598-018-34778-w)
Supplement: Supplementary file 1 — Supplementary Information [file 41598_2018_34778_MOESM1_ESM.docx]

**On the expedient solution of the magneto-hydrodynamic Jeffery-Hamel flow of Casson fluid**

S. S. Nourazar^1,*^, A. Nazari-Golshan^2^ and F. Soleymanpour^1^

*^1^Mechanical Engineering Department, Amirkabir University of Technology, Tehran, Iran*

*^2^Physics Department, Shahed University, Tehran, Iran*

**Appendix**

The Fourier transform of the function$f(x)$may be defined by the following function:

$$\mathrm{F}(\omega)=\int_{-\infty}^{\infty} f(x)\mathrm{e}^{-i\omega x}dx$$

and its inverse transform is given by:

$$f(x)=\int_{-\infty}^{\infty} \mathrm{F}(\omega)\mathrm{e}^{i\omega x}d\omega$$

where the independent variables$x$and$\omega$ represent space coordinate and frequency respectively.
